# Are Hox Genes Ancestrally Involved in Axial Patterning? Evidence from the Hydrozoan *Clytia hemisphaerica* (Cnidaria)

**DOI:** 10.1371/journal.pone.0004231

**Published:** 2009-01-21

**Authors:** Roxane Chiori, Muriel Jager, Elsa Denker, Patrick Wincker, Corinne Da Silva, Hervé Le Guyader, Michaël Manuel, Eric Quéinnec

**Affiliations:** 1 UPMC Univ Paris 06, UMR 7138 CNRS UPMC MNHN IRD, Case 05, Paris, France; 2 Sars International Centre for Marine Molecular Biology, Bergen, Norway; 3 Genoscope, Centre National de Séquençage, Evry, France; Michigan State University, United States of America

## Abstract

**Background:**

The early evolution and diversification of Hox-related genes in eumetazoans has been the subject of conflicting hypotheses concerning the evolutionary conservation of their role in axial patterning and the pre-bilaterian origin of the Hox and ParaHox clusters. The diversification of Hox/ParaHox genes clearly predates the origin of bilaterians. However, the existence of a “Hox code” predating the cnidarian-bilaterian ancestor and supporting the deep homology of axes is more controversial. This assumption was mainly based on the interpretation of Hox expression data from the sea anemone, but growing evidence from other cnidarian taxa puts into question this hypothesis.

**Methodology/Principal Findings:**

Hox, ParaHox and Hox-related genes have been investigated here by phylogenetic analysis and *in situ* hybridisation in *Clytia hemisphaerica*, an hydrozoan species with medusa and polyp stages alternating in the life cycle. Our phylogenetic analyses do not support an origin of ParaHox and Hox genes by duplication of an ancestral ProtoHox cluster, and reveal a diversification of the cnidarian HOX9-14 genes into three groups called A, B, C. Among the 7 examined genes, only those belonging to the HOX9-14 and the CDX groups exhibit a restricted expression along the oral-aboral axis during development and in the planula larva, while the others are expressed in very specialised areas at the medusa stage.

**Conclusions/Significance:**

Cross species comparison reveals a strong variability of gene expression along the oral-aboral axis and during the life cycle among cnidarian lineages. The most parsimonious interpretation is that the Hox code, collinearity and conservative role along the antero-posterior axis are bilaterian innovations.

## Introduction

Since the discovery of mice and *Drosophila* Hox clusters [1]–[3] the evolutionary conservation of the Hox axial patterning system has been the starting point of a conceptual framework in evolutionary developmental biology (evo-devo). The fact that orthologous genes display similar genomic organisation and expression patterns with comparable spatial and temporal characteristics in distantly related species has provided clues for understanding the evolution of the body plan. Indeed major morphological changes during animal evolution, and notably those involved in the edification of the body plan, are intimately associated with modified Hox gene expression patterns and assigned to changes affecting developmental regulatory networks (acquisition, loss or co-option of functionalities) [4]–[7]. A hierarchical categorisation of variation in Hox pathways has been proposed to be connected to the hierarchy of taxonomic levels [8]. Each phylum could hence be characterised by a particular Hox pattern responsible for the establishment of its specific body plan. This particular pattern establishes a “Hox code” consisting in a combinatorial information of position along the antero-posterior axis [9].

This key concept has led authors to try reconstructing the ground pattern of the bilaterian last common ancestor [10]–[12]. Hox genes and their conserved collinear expression are hence believed to be part of the archetypal developmental genetic tool-kit of Urbilateria (e. g. [13]–[14]). The ParaHox cluster, the hypothetical evolutionary sister of the Hox cluster [15], is also supposed to be part of this ancestral tool-kit, being implicated in endoderm patterning whereas Hox genes are more specifically expressed in ectoderm [16]. Under this hypothesis, body plan evolution would be closely linked to the genomic organisation and expression of the Hox/ParaHox gene family.

The role of Hox genes in patterning the antero-posterior axis is strikingly conserved among bilaterians in spite of a huge diversification of body plans, but the situation appears much more complex outside Bilateria. As cnidarians were shown to be the sister-group of the Bilateria [17]–[18], they can give crucial clues on the evolution of Hox/ParaHox genes, in particular to test the origin of the Hox code patterning system. The Cnidaria constitute a widely diversified taxon with a quite unified organisation. They share a unique body plan with a single polarity axis (the oral-aboral axis) but exhibit various life cycles, comprising a pelagic (polyp) or a benthic form (medusa) or both alternating. The Cnidaria encompass five main taxa [19]: the Anthozoa (corals, sea anemone), Staurozoa, Cubozoa, Scyphozoa and Hydrozoa. Anthozoans are the sister group to the remaining cnidarians, which form together the medusozoans. Hox and ParaHox genes have been identified from various cnidarian species [20]–[30]. Expression patterns of a number of genes have also been investigated [24], [27], [29], [31]–[37]. The interpretation of these data have led authors to contradictory conclusions about the early evolution of the Hox/ParaHox family and of their functions in relation to axial polarity.

The Hox/ParaHox family was undoubtedly already present and diversified in the cnidarian / bilaterian ancestor [25]–[30]. However recent studies have upheld conflicting views about the composition of the cnidarian ancestral gene complement. Based on phylogenetic relationships between cnidarian and bilaterian sequences, most writers agree on the existence of true Hox genes in cnidarians (e.g. [29], [34], [37], [38]), even if a recent study claimed the contrary [35]. There is also general agreement that the common cnidarian / bilaterian ancestor possessed “anterior” Hox (HOX1 and HOX2 paralogy groups) and ParaHox (GSX) genes, but lacked HOX3 and “median” (HOX4-8) Hox genes (e.g. [29], [34], [37], [39]). On the contrary, the existence of “posterior” genes is more controversial, different authors supporting their presence [29], [34], [37] or absence [35], [38] in cnidarians. These divergent interpretations imply incompatible evolutionary scenarios: either the cnidarian/bilaterian ancestor possessed both “anterior” and “posterior” Hox-like genes, or “non-anterior” genes result from independent diversification in the bilaterian and cnidarian lineages. The phylogenetic analyses discussed in these contradictory studies often include few cnidarian taxa [35], [37] and a reduced or absent outgroup of non-Hox/ParaHox genes [33], [35], [38].

In addition, while a Hox code was almost certainly operating in the bilaterian ancestor, the possible implication of cnidarian Hox genes in a similar system remains unclear. Most studies have interpreted Hox genes pattern restricted along the oral-aboral axis as probably reflecting a role of cnidarian Hox genes in axial patterning [29], [31], [33]–[35], [37]. Expression data in the sea anemone (Anthozoa) have even led to the conclusion that the bilaterian antero-posterior and the cnidarian oral-aboral axes are homologous [34]. Concomitantly, Hox expression patterns in the sea anemone have been used as a clue to advocate the existence of a Hox code in cnidarians and in the cnidarian / bilaterian ancestor [37]. However, expression data from other cnidarians (particularly the hydrozoans *Podocoryne* and *Eleutheria*) cast doubt on the conservation of a Hox code in cnidarians [35]. To uncover the characteristics of Hox gene expression in the cnidarian ancestor (a prerequisite for high-level comparisons with the bilaterians) more data from various cnidarian species are needed.

We have isolated Hox-related genes in *Clytia hemisphaerica*, a hydrozoan (Hydroidolina, Thecata) species that possess both medusa and polyp stages, and investigated the diversity of expression patterns during development and at the medusa stage. Phylogenetic analyses have revealed instances of gene gain and loss in the various cnidarian lineages and highlighted a diversity of evolutionary histories among them. We have compared the expression of Hox and ParaHox orthologues among cnidarians and reconsidered the possible implication of cnidarian Hox genes in axial patterning through a Hox code. Altogether, these results allow a reappraisal of which characteristics are ancestral with respect to the bilaterians and which ones are bilaterian novelties.

## Results

### The *Clytia* Hox/ParaHox-extended complement is representative of the cnidarian phylogenetic diversity

**Figure 1:**
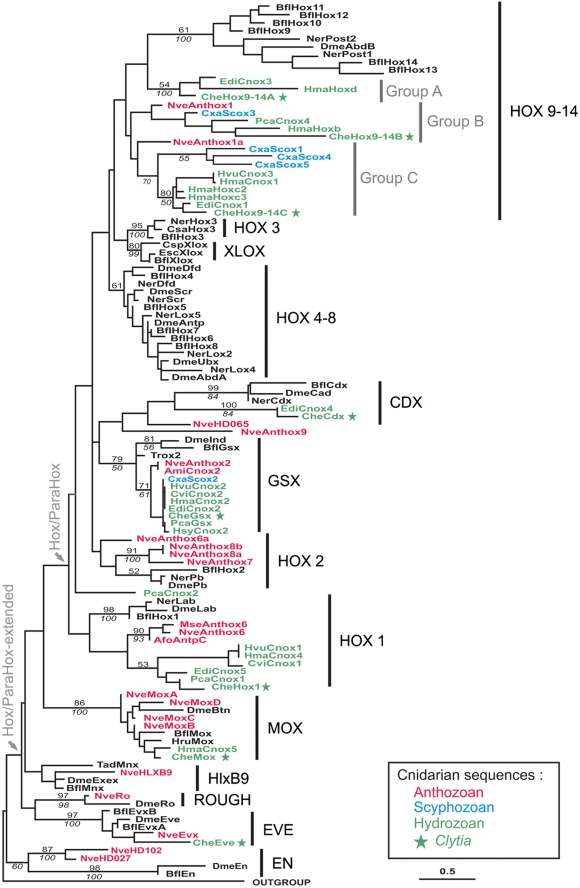
Phylogenetic relationships between cnidarian, placozoan and bilaterian Hox/ParaHox related homeodomains inferred by ML analysis. Support values higher than 50% for each Hox/ParaHox related group are shown on the branches. Numbers above the branches indicate ML bootstrap values (100 replicates). Numbers below the branches indicate NJ bootstrap values (1000 replicates). Abbreviations: Ami, *Acropora millepora*; Bfl, *Branchiostoma floridae*; Che, *Clytia hemisphaerica*; Csa, *Cupiennus salei*; Csp, *Capitella sp.*; Cvi, *Chlorohydra viridissima*; Cxa, *Cassiopeia xamachana*; Dme, *Drosophila melanogaster*; Edi, *Eleutheria dichotoma*; Esc, *Euprymna scolopes*; Hma, *Hydra magnipapillata*; Hru, *Haliotis rufescens*; Hsy, *Hydractinia symbiolongicarpus*; Hvu, *Hydra vulgaris*; Mse, *Metridium senile*; Ner, *Nereis virens*; Nve, *Nematostella vectensis*; Pca, *Podocoryne carnea*; Tr, *Trichoplax adhaerens*.

Sixteen ANTP homeodomain sequences have been retrieved by tBLASTn search from our *Clytia* EST collection (figures S1 and S2). Among them, 8 belong to the Hox-extended family, which includes Hox, ParaHox, Mox, HlxB9, Rough and Eve genes (figure 1). The Hox/ParaHox-extended complement retrieved here from *Clytia* equates in gene number the complement present in the full genomic sequence of *Hydra* (8 genes) but is less rich than the repertoire present in the sea anemone genome (15 genes) [38].

This *Clytia* Hox/ParaHox complement well represents the diversity generally encountered in this gene family in cnidarian species. All cnidarian Hox or ParaHox groups contain at least one sequence from *Clytia*, except HOX2 (figure 1). Among the 8 Hox/ParaHox clades identified in our tree, the “anterior” (HOX1 and HOX2 / GSX) and “posterior” (HOX9-14 / CDX) groups contain cnidarian sequences, but sequences from *Clytia* or other cnidarian species are absent from the “median” Hox and ParaHox groups (HOX3, HOX4-8, XLOX), as previously noticed (e.g. [29]). Hence, the Hox/ParaHox sequences from *Clytia* are distributed as follows, in both the ML and the NJ trees (figures 1 and S2): one sequence in the HOX1 “anterior” Hox group, subsequently named *CheHox1*, three in the “posterior” Hox HOX9-14 group named *CheHox9-14A*, *CheHox9-14B* and *CheHox9-14C*, one in the GSX “anterior” ParaHox group named *CheGsx* and one in the “posterior” ParaHox group CDX named *CheCdx*. In addition, a *CheMox* sequence and a *CheEve* sequence were also identified.

Lack of statistical robustness is a classical difficulty when inferring tree with short sequences, and this is particularly true of homeodomain sequences (only 60 amino-acids). In addition, it has been shown that bootstrap values are not reliable robustness estimators for data sets containing less than 100 characters (notably, “true clades” might be unsupported by bootstraps, [40]), and paucity of characters is an intrinsic limitation of this kind of data sets for which there is no solution. Thus, our tree contains very few statistically supported branches; notably most of the deepest nodes have bootstrap values lower than 50% (figures S1 and S2).

### Among Hox/ParaHox groups, HOX9-14 contains the highest diversity of cnidarian sequences

The wide range of cnidarian sequences integrated in our analysis highlights the diversity of cnidarian HOX9-14 genes and their complex phylogenetic relationships with bilaterian sequences. Cnidarian sequences related to bilaterian “posterior” Hox (HOX9-14) branch basally to the latter in paraphyly. Thus, the HOX9-14 group is organised in four sub-groups: one bilaterian and three cnidarian sub-groups which we propose to call Group A, Group B and Group C (figure 1). The statistically supported Group A is the sister-group to bilaterian sequences.

Interestingly the ParaHox groups classically defined as “anterior” (GSX) or “posterior” (CDX) do not appeared phylogenetically related to the so-called “anterior” or “posterior” Hox groups, in contradiction with some of the former studies [15]. In our global analysis of cnidarian and bilaterian data, GSX and CDX are sister-groups (figure 1). Four cnidarian homeodomains are related to the bilaterian ParaHox sequences CDX. They are branched in paraphyly, with no statistical support and rather long branches. This result is in accordance with previous studies (e.g. [29], [37]) except for *NveAnthox9* (also named *NVHD117* in [41] or *HoxR* in [38]) which has been previously described as a possible pseudogene [37].

Globally, orthologies of the cnidarian sequences with their bilaterian counterparts are clearer for the “anterior” groups HOX1 and HOX2 (Hox) and GSX (ParaHox) than for the “posterior” Hox and ParaHox groups, cnidarian sequences branching in paraphyly in the latters.

### Diversification of Hox/ParaHox complements among cnidarian lineages

By including genes from various cnidarian species belonging to Anthozoa, Scyphozoa and Hydrozoa, our analysis allows to identify lineage-specific gene duplications or losses through the comparison of the topology within the gene tree with the known phylogenetic relationships between included species [19], [42]–[43].

In some cases, gene relationships among cnidarian groups are congruent with the species phylogeny: hydrozoan sequences are sister-group to scyphozoan sequences, with anthozoan sequences branching basally to this ensemble. This occurs among the cnidarian GSX and HOX9-14 B and C groups (figure 1).

In contrast, some cnidarian lineages are lacking in several gene groups. Hence HOX2 genes are absent from the *Hydra magnipapillata* full genome and have not been identified until now in other hydrozoan or scyphozoan species, being only known from the anthozoan *Nematostella* (figure 1). This can be taken as an indication that HOX2 genes were lost at some time during the history of the medusozoans. Similarly, anthozoans have probably lost their HOX9-14A gene, a group that contains only hydrozoan sequences (figure 1). Although scyphozoan sequences are lacking in the HOX1, HOX2, HOX9-14A and CDX groups (figure 1), these absences could be due to non exhaustive sampling from PCR surveys, a full genome sequence being currently lacking for this cnidarian lineage [28].

### *Clytia* HOX9-14 genes are expressed in opposite domains along the oral-aboral axis during development

**Figure 2:**
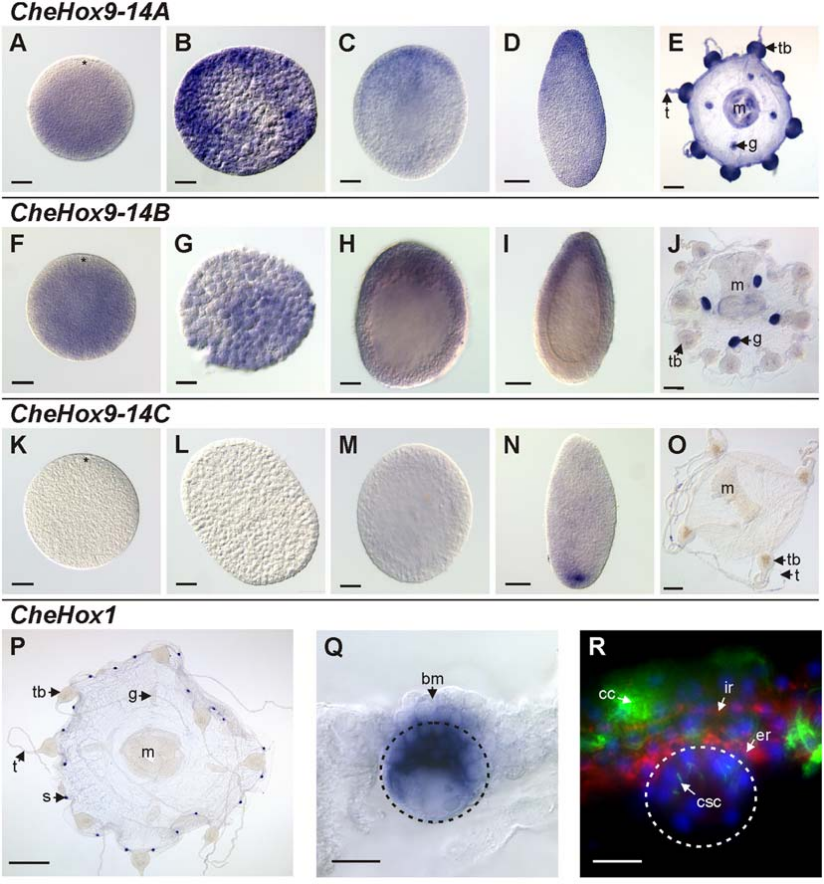
Developmental and medusa-specific expression of Hox genes in *Clytia hemisphaerica*. A–E: *CheHox9-14A* expression; A: non-fertilised egg with animal pole on the top; B: blastula; C: gastrula with ingression pole ( = animal and future oral pole) on the top; D: one-day-old planula with oral/posterior pole on the top; E: medusa. F–J: *CheHox9-14B* expression; F: non-fertilised egg with animal pole on the top; G: blastula (animal pole on the top); H: gastrula with ingression pole ( = animal and future oral pole) on the top; I: one-day-old planula with oral/posterior pole on the top; J: medusa. K–O: *CheHox9-14C* expression; K: non-fertilised egg with animal pole on the top; L: blastula (animal pole on the top); M: gastrula with ingression pole on the top; N: one-day-old planula with oral/posterior pole on the top; O: medusa. P–R: *CheHox1* expression; P: general view of the adult medusa; Q: higher magnification of the statocyst of the medusa delineated by the dotted line; R: Statocyst structure (delineated by dotted line) highlighted by immunohistochemistry, with dapi staining of nucleus in blue, anti-acetylated-α-tubulin immunostaining of cilia in green and anti-FMRF-amide immunostaining of nerve cells in red. Scale bars: P: 500 µm; E, J, O: 100 µm; A–D, F–I, K–N: 50 µm; Q, R: 10 µm. Legends: bm: bell margin; cc: cilia of the circular canal digestive cells; csc: cilia of the statocyst sensory cells; er: external nerve ring; g: gonad; ir: internal nerve ring; m: manubrium; s: statocyst; t: tentacle; tb: tentacle bulb.

The three *Clytia* genes related to bilaterian HOX9-14 are all expressed during development. However, they exhibit highly distinct expression domains along the oral-aboral axis and differing dynamic characteristics in the course of the life cycle (figure 2A–O).

*CheHox9-14A* is expressed throughout development (figure 2A–E). Transcripts are firstly detected in the unfertilised eggs in the whole cytoplasm but they are absent from the area surrounding the nucleus, corresponding to the animal pole and to the future aboral end of the polyp (figure 2A). *CheHox9-14A* maternal transcripts segregate in subsets of cells (data not shown). Consequently the expression in the blastula is restricted to subgroups of cells without clear orientation (figure 2B). At the onset of gastrulation, expressing cells are localised in the oral hemisphere, where ingression takes place (figure 2C). After gastrulation, in the 1-day old planula, transcripts are dispersed throughout the ectoderm with a higher concentration at the posterior/oral pole (figure 2D). At the medusa stage *CheHox9-14A* has a maternal expression detected in the maturing oocytes of the female gonads (figure 2E). This gene exhibits also a somatic expression throughout the ectoderm of the tentacle bulbs and in the manubrium (figure 2E).

The expression pattern of *CheHox9-14B* is very similar to that of *CheHox9-14A* (figure 2F–J). However transcripts seem not to be excluded from the nucleus area (figure 2F). During later developmental stages, *CheHox9-14A* and *CheHox9-14B* expression profiles are undistinguishable (figure 2G–H compared with 2B–C). In the 1-day-old planula, *CheHox9-14B* mRNA are restricted to the posterior/oral half of the larva (figure 2I). At the medusa stage, *CheHox9-14B* is only expressed in the maturing oocytes and no somatic expression is detected (figure 2J).

The expression of *CheHox9-14C* is much more temporally restricted during the life cycle (figure 2K–O). No expression has been observed during early development (figure 2K–M) and signal is firstly detected in the 1-day-old planula (figure 2N). At this stage transcripts are localised at the anterior/aboral pole, in a few ectodermal cells (figure 2N). No signal has been detected at the medusa stage (figure 2O).

### The *Clytia* HOX1 gene is not expressed along the oral-aboral axis but specifically in medusa sensory organs

Expression of *CheHox1*, the only “anterior” Hox gene known from *Clytia*, was only detected at the medusa stage (figure 2P–Q) while no expression has been observed during development or in the planula (not shown). *CheHox1* mRNA are specifically localised in the statocysts (figure 2P), the equilibration organs regularly arranged on the bell rim of the medusa. The *CheHox1* expressing cells are localised in the basal epithelium of the statocyst, near the bell margin (figure 2Q). According to this localisation they are interpreted as ciliated mechano-sensory cells. Hence, *Clytia* statocysts are ectodermal derivatives consisting in a closed pocket limited on the distal side by a thin epithelium and on the proximal side (near the bell margin) by a monociliated sensory epithelium (figure 2R) expressing *CheHox1* (figure 2Q).

### The *Clytia* GSX gene is exclusively expressed at the medusa stage whereas *CheCdx* is also expressed during development

**Figure 3:**
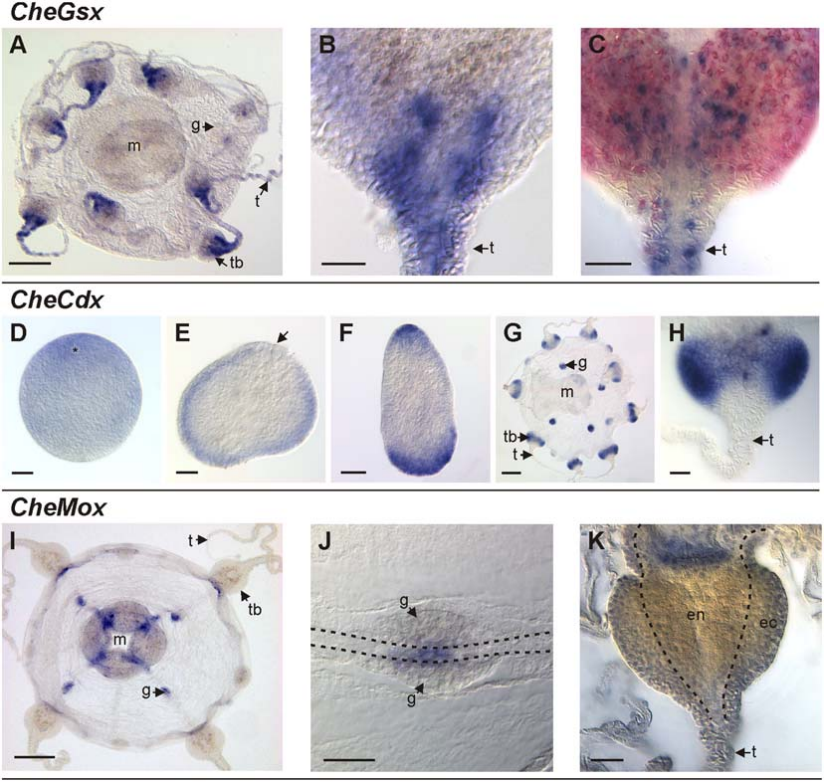
Expression of ParaHox and Mox genes in *Clytia hemisphaerica*. A–C: *CheGsx* expression; A: general view of the medusa; B: higher magnification of the distal part of the tentacle bulb; C: distal part of the tentacle bulb after double *in situ* hybridisation with *CheGsx* (in blue) and *CheMcol3-4* (in red) riboprobes. D–H: *CheCdx* expression; D: unfertilised egg with animal pole on the top; E: gastrula with ingression pole ( = animal and future oral pole) on the top (arrow); F: expression in one-day-old planula with oral/posterior pole on the top; G: general view of the medusa; H: higher magnification of the tentacle bulb. I–K: *CheMox* expression; I: general view of the medusa; J: higher magnification of the radial canal (delineated by dotted lines) crossing the gonad; K: higher magnification of the tentacle bulb showing the ectodermal and endodermal layers separated by a dotted line. Scale bars: A, G, I: 100 µm; B, C, H, J, K: 20 µm; D–F: 50 µm. Legends: ec: ectoderm; en: endoderm; g: gonad; m: manubrium; t: tentacle; tb: tentacle bulb.

The ParaHox gene *CheGsx* is expressed specifically at the medusa stage (figure 3A) and no expression has been detected at other stage during the life cycle (not shown). *CheGsx* transcripts are localised in scattered cells in the tentacles and in the tentacle bulbs (figure 3A), spherical enlargements on the bell margin that bear tentacles. The tentacle bulb is a specialised region devoted to the continuous production of tentacle cells, the latter being permanently used and destroyed because of prey capture. This structure has been recently shown to be a site of intensive nematogenesis characterised by an ordered progression of cell stages along its proximo-distal axis [44]. Nematocyte progenitors are localised in the proximal region of the bulb, near the bell margin, and the nematoblasts move during their differentiation towards the tentacle, where they maturate. The *CheGsx*-expressing cells are not homogeneously distributed along the proximo-distal axis of the tentacle bulb. They form, in the ectodermal layer, isolated basi-epithelial spots concentrated in the more distal part of the bulb and in the tentacle base, and also more concentrated on the abaxial side of the bulb (figure 3B). This position does not correspond to the crescent-shaped distribution of nematocyte precursors, but rather to the neuron and sensory cell-rich area of the bulb ectoderm. Furthermore, *CheHox1* is not co-expressed with the minicollagen *CheMcol3-4* (figure 3C), a nematocyte capsule structural component expressed during differentiation of the tentacle main nematocyte type [44]. In addition, we have failed to identify nematocyte capsules (easily distinguishable using DIC optics) inside the *CheGsx* expressing cells (figure 3B–C). Thus, *CheGsx* is probably expressed in neural cells or precursors rather than in nematoblasts.

Contrary to *CheGsx*, the other *Clytia* ParaHox gene *CheCdx* is expressed during development (figure 3D–F). Staining is observed in unfertilised eggs around the nucleus at the animal pole (figure 3D). Expression is maintained after fertilisation and during cleavage (not shown). When gastrulation starts, *CheCdx* transcripts are observed in the whole embryo, except at the oral pole (figure 3E). In the 1-day old planula they are concentrated in the ectoderm at both the oral and aboral poles (figure 3F).

The *Clytia* CDX ortholog is also expressed at the medusa stage where transcripts are concentrated in maturing oocytes of the female gonads and in tentacle bulbs (figure 3G). In the latter, *CheCdx* positive cells are located in the ectodermal layer and are more densely packed than *CheGsx* expressing ones (figure 3H). They form a crescent shaped pattern in a median position along the poximo-distal axis, interrupted on the external side of the bulb. This expression is more extended and proximal than that of *CheGsx* and identical to that of dickkopf-3 (*CheDkk-3*) or minicollagens (*CheMcol3-4*), as previously described [44]. The *CheCdx* expression pattern in tentacle bulbs is thus compatible with a localisation in differentiating nematoblasts.

### The *Clytia* MOX gene is expressed in restricted areas of the medusa endoderm

*CheMox* is exclusively expressed at the medusa stage (figure 3I) and no transcripts have been detected at other stages (not shown). *CheMox* expression is restricted to endodermal tissues, in particular areas of the gastrovascular system. Hence *CheMox* transcripts have been detected in the manubrium in four regions adjacent to the radial canals (figure 3I). *CheMox* expressing cells are also present in the radial canals against the gonads (figure 3J) and in the ring canal near the tentacle bulbs (figure 3K).

## Discussion

### The complex history of cnidarian Hox genes and its bearing on early Hox evolution

**Figure 4:**
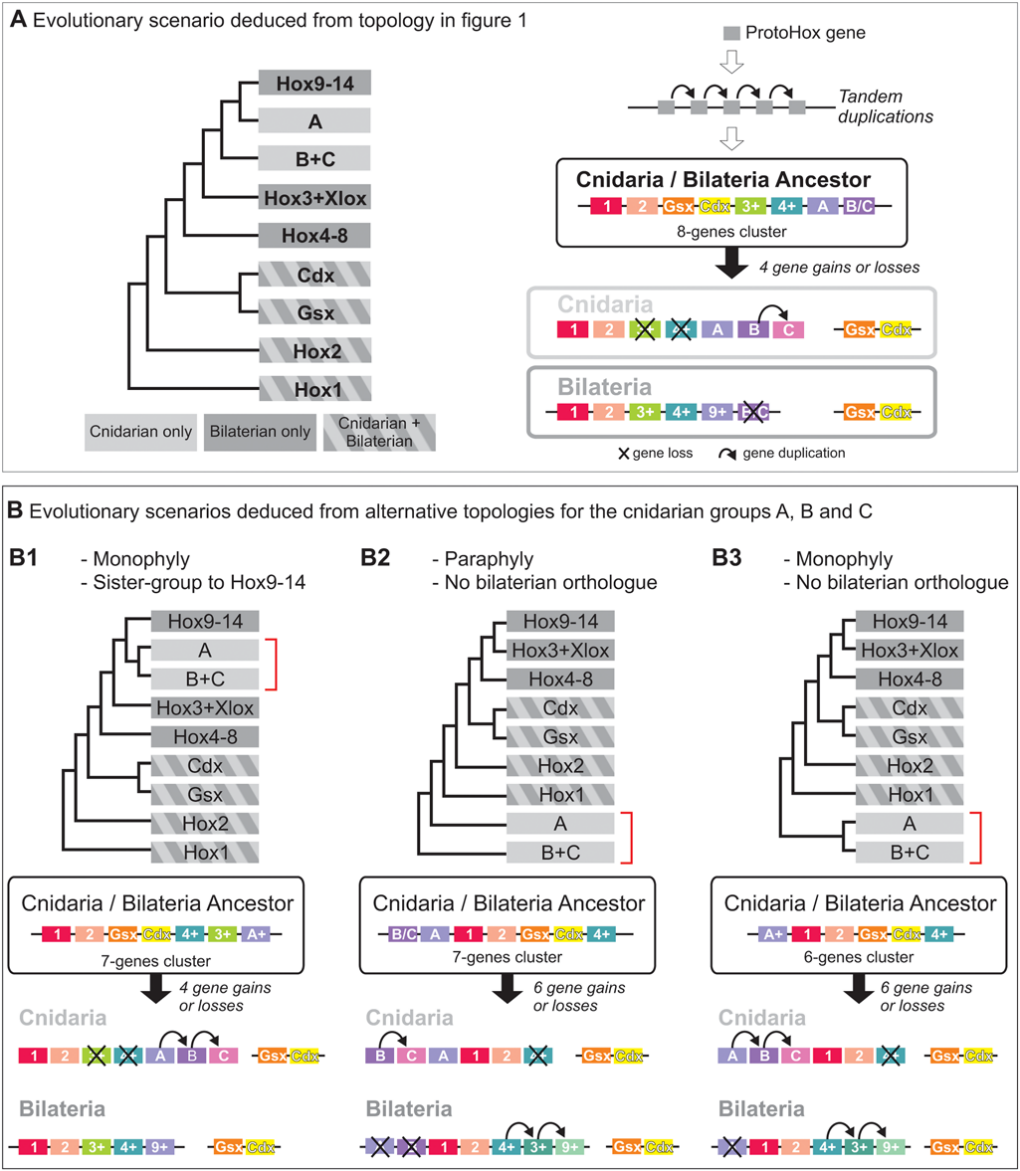
Evolutionary origin of cnidarian and bilaterian Hox/ParaHox complements. A: Evolutionary scenario (on the right) inferred from phylogenetic relationships between cnidarian and bilaterian Hox and ParaHox genes (on the left, simplified topology of figure 1). Cnidarian groups A, B and C branch in paraphyly in respect to bilaterian “posterior” Hox genes (Hox9-14) and form a cnidarian “posterior” Hox group. B: Evolutionary scenarios deduced from alternative phylogenetic position for the cnidarian “posterior” Hox genes (groups A, B and C from figure 4A). B1: A, B and C form a monophyletic group orthologue to bilaterian “posterior” Hox genes (Hox9-14); B2: A, B and C branch in paraphyly in respect to all other cnidarian and bilaterian Hox/ParaHox genes, with thus no orthology with a particular bilaterian Hox or ParaHox group; B3: A, B and C form a monophyletic group orthologous to all other cnidarian and bilaterian Hox/ParaHox genes but not to a particular Hox or ParaHox group.

Our rooted analysis of the Hox-extended family (figure 1) agrees with previous studies [45]–[49] concerning the presence of true Hox genes in cnidarians and bilaterians and their probable absence from sponges, ctenophores and placozoans, leading to the conclusion that this gene family originated in an exclusive cnidarian / bilaterian ancestor, or was lost in other metazoan lineages [50]. Also consistent with previous analyses, the “anterior” HOX1 and HOX2 groups have clear cnidarian orthologues, while “median” genes (HOX3-8) are on the contrary absent from cnidarian genomes (figure 1; [29], [34], [37]–[38]). For the latter, our topology suggests an origin before cnidarian / bilaterian divergence and subsequent losses of HOX3/XLOX and HOX4-8 in cnidarian lineages, albeit without statistical support (figure 4A).

Thanks to the integration of a wide range of cnidarian taxa, our tree highlights a diversification of cnidarian “posterior” Hox genes that was not previously noticed. We recovered a monophyletic HOX9-14 group comprising cnidarian genes organised in three groups (called here group A, B and C) arranged in paraphyly with respect to bilaterian HOX9-14 (figure 1). This topology suggests that the cnidarian / bilaterian ancestor possessed two or three posterior Hox-like genes, only one (related to cnidarian HOX9-14 group A) being retained in the bilaterian lineage (figure 4A). However, given the lack of statistical support of the tree, alternative topologies cannot be excluded. These hypotheses imply differences in the ancestral Hox/ParaHox complement and in lineage-specific gene gains or losses. Hence three main topologies, and their corresponding inferred evolutionary scenarios, must be considered (figure 4B). First of all, cnidarian groups A, B and C may form a monophyletic group, instead of a paraphyletic one, orthologous to bilaterian HOX9-14 (figure 4B-1). This would imply an ancestral Hox complement with only one “posterior” Hox-like gene, the diversification of HOX9-14 occurring further independently in cnidarians and bilaterians. This “monophyly” hypothesis (figure 4B-1) requires the same number of gene gains or losses as the “paraphyly” hypothesis sustained by our phylogeny (figure 4A), both being thus equally parsimonious. Secondly the position of cnidarian sequences related to bilaterian HOX9-14 may result from long branch attraction artefact between rapidly evolving sequences. Hence, cnidarian A, B and C groups could rather branch in paraphyly (figure 4B-2) or in monophyly (figure 4B-3) as sister-group to the whole Hox/ParaHox clade. This would imply a reduced ancestral Hox complement without true “posterior” Hox-like genes and the presence of at least one ancestral Hox/ParaHox-like gene further lost in the bilaterian lineage (figure 4B-2 and 4B-3). These hypotheses necessitate a higher number of bilaterian-specific gene duplications giving rise to HOX9-14, HOX3/XLOX and HOX4-8, and are hence less parsimonious than the former ones (figure 4A and B-1). Furthermore the absence of cnidarian orthologues of the bilaterian “posterior” (HOX9-14) genes has been sustained by some authors [35], [38], but these assumptions were based on non-rooted (neighbour-net method in [38]) or poorly rooted [35] analyses. Hence, we consider the existence of cnidarian Hox genes orthologue to bilaterian HOX9-14 as the most reliable hypothesis (figure 4A), postulating an ancestral Hox complement with two “anterior” Hox genes (one HOX1-like and one HOX2-like) and one to several “posterior” Hox genes (HOX9-14-like).

Important events of gene loss or duplication affected Hox genes later on during the evolution of the Cnidaria, leading to a diversification of the gene sets among cnidarian lineages. For example, hydrozoans have lost their HOX2 genes (present in anthozoans; inconclusive data for scyphozoans; figure 1). In turn, anthozoan species seem to have lost their group A HOX9-14 gene (present in hydrozoans; inconclusive data for scyphozoans; figure 1). Furthermore, each major cnidarian lineage experienced specific duplications: group C HOX9-14 genes were independently duplicated in the Scyphozoa and in the Hydrozoa, and several duplications increased the number of HOX2 genes in the Anthozoa (figure 1). An important consequence is that no single cnidarian species can be taken as representative of the cnidarian ancestor in terms of the Hox gene complement.

### Hox gene expression data in *Clytia* and other cnidarians do not support the conservation of a “Hox code”

The proposal that cnidarian Hox genes have a role in patterning the oral-aboral axis, reminiscent of the “Hox code” conserved among bilaterian species, was initially prompted by the direct comparison of Hox expression patterns obtained in *Nematostella* (Anthozoa) with what is known of their orthologues in bilaterian species [34], [37]. Hox expression was claimed to be collinear in the sea anemone and to support homology between the cnidarian oral end and the bilaterian head [51]. Thanks to the availability of data concerning other cnidarian species, it becomes now feasible to address the role of Hox genes in the common ancestor of Cnidaria, before extending the comparison to the more distantly-related Bilateria, a task for which two distinct levels of interrogation should be distinguished. First, is Hox gene expression in cnidarian species collinear, as expected of a cnidarian “Hox code”? The second pivotal question is whether or not there is conservation, among the major cnidarian lineages, of the region along their main body axis where a given Hox orthologue is expressed, as expected if cnidarian Hox genes have a conserved role in patterning this axis.

**Figure 5:**
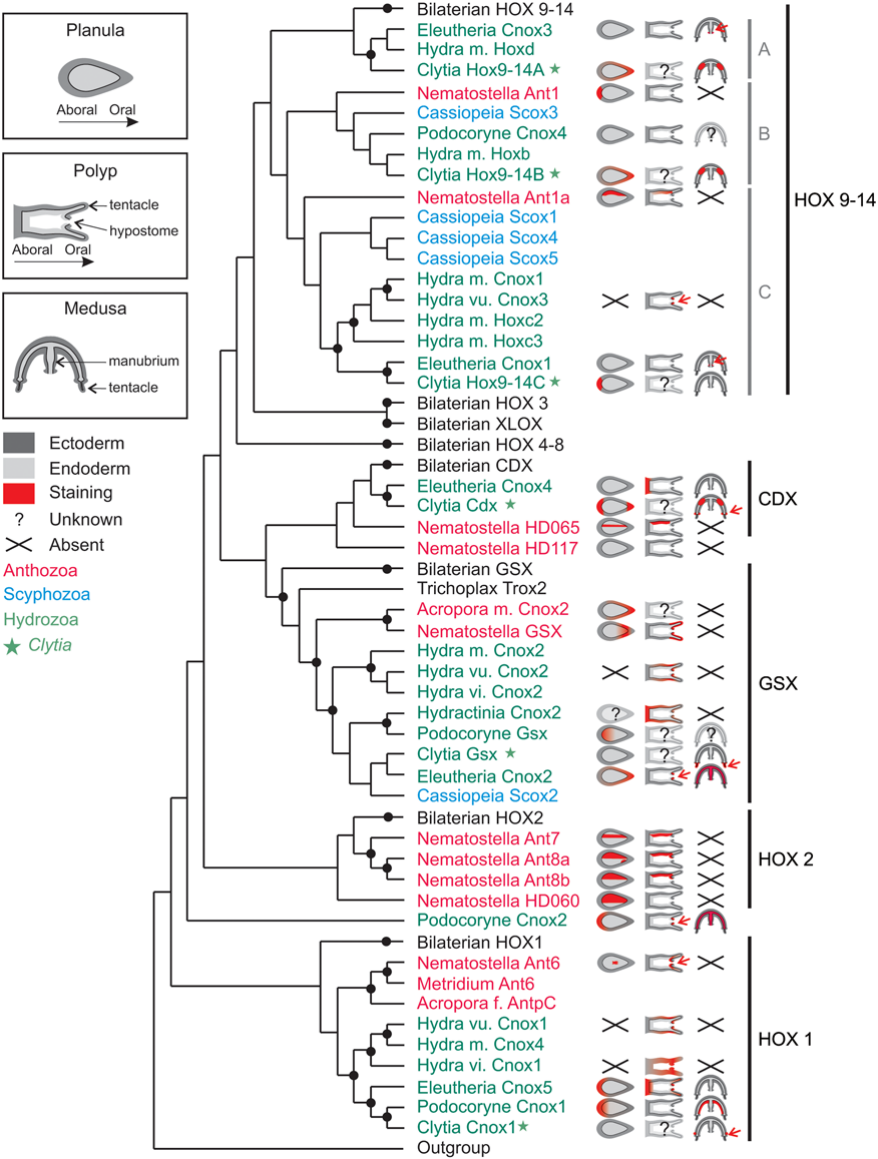
Comparison of Hox expression patterns among cnidarian species. Each expression pattern is represented by a red shading on the planula, polyp or medusa diagrams. The diagrams illustrate a schematic view of each stage after theoretical longitudinal section to expose the ectodermal layer (dark gray) and the endodermal layer (light gray) separated by the mesoglea (black line). All published expression data for cnidarian species were mapped on the ML phylogenetic tree. Branches for bilaterian sequences were compressed to simplify the figure. Black disks indicate statistically supported nodes.

Collinearity has been initially defined for non-fragmented Hox clusters as a correlation between the physical order of Hox genes in the genome and their expression domains along the antero-posterior axis of bilaterian animals [52], “cis-collinearity” according to Duboule [53]. However, in the case of a partially or totally dispersed cluster or when no genomic data are available, Hox expression domains along the antero-posterior axis can be correlated with the phylogenetic position of the genes with respect to paralogous groups in species with an intact cluster (“trans-collinearity” according to Duboule [53]). The only reported instance of a genomic linkage between several Hox genes in Cnidaria concerns the *Nematostella* genome, which contains a 50 kb cluster of five genes arranged in the following order: the HOX1 gene *NveAnthox6*, the EVE gene *NveEve* and the three HOX2 genes *NveAnthox8b*, *NveAnthox8a* and *NveAnthox7* [38]. However, the expression of these four Hox genes along the oral-aboral axis shows no evidence of cis-collinearity, *Anthox6* being expressed in the pharyngeal endoderm and *NveAnthox8a-8b-7* being expressed all along the axis in the body wall endoderm (figure 5; [37]). For the remaining cnidarian species (and for the remaining *Nematostella* genes), lack of physical linkage or of information about it leaves trans-collinearity (with the order of orthologous Hox gene expression in bilaterians taken as a reference) as the only potential form of collinearity to be considered.

The expression of *Clytia* Hox genes in the planula larva reported here (figure 2) is clearly not trans-collinear. The “anterior” HOX1 gene *CheHox1* is apparently not expressed in the larva. “Posterior” (HOX9-14) genes have restricted expression sites along the oral-aboral axis of the planula, but with two paralogues (*CheHox9-14A* and *B*) mainly expressed at the oral pole and the third one (*CheHox9-14C*) expressed at the opposite aboral pole (figure 2), a situation clearly incompatible with collinearity. Total absence of trans-collinearity was previously reported for the hydromedusae *Eleutheria* (in which Hox genes are physically dispersed; [35]) and *Podocoryne* (no genomic data), since their HOX1 and HOX9-14 genes are not expressed at the same stage of the life cycle (figure 5; [33], [35]). In contrast, expression of “anterior” vs. “posterior” genes in different domains along the oral-aboral axis, potentially evoking trans-collinearity, has been reported in *Nematostella* (*NveAnthox6* (HOX1) and *NveAnthox1* (HOX9-14) expressed at opposite poles; figure 5; [34]) and more arguably in *Hydra* (with a difference in the extension along the polyp axis of the overlapping expression domains of *HvuCnox1* (HOX1) and *HvuCnox3* (HOX9-14); figure 5; [29]). It must be noticed that in *Nematostella*, the HOX2 genes (*NveAnthox7*, *NveAnthox8a*, *NveAnthox8b*) and the other HOX9-14 gene (*NveAnthox1a*) are widely expressed all along the oral-aboral axis in the body wall endoderm (figure 5; [37]) and are hence excluded from the potential “trans-collinearity”.

Under the hypothesis of a conserved role for Hox genes in patterning the cnidarian main body axis, not only their expression should be collinear, but orthologous Hox genes from different cnidarian species are expected to be expressed in similar domains along the oral-aboral axis. This is clearly not the observed situation when expression data from various cnidarian taxa are compared. Firstly, in some cases orthologous genes are not expressed at the same stage of the life cycle. For example, the *Clytia* HOX9-14 group A gene *CheHox9-14A* is expressed at the oral pole of the planula (figure 2D) whereas its counterpart in an other hydrozoan, *Eleutheria* (*EdiCnox3*), is only expressed in the medusa (figure 5; [35]). The *Clytia* HOX9-14 group C gene *CheHox9-14C*, expressed at the aboral pole of the planula, is orthologous to *Eleutheria Cnox1*, which has no detected expression at the planula stage. In addition, when orthologous genes are expressed at the same stage, their transcripts are often localised at opposite poles along the oral-aboral axis. Hence, among HOX9-14 group B genes, *CheHox9-14B* is expressed at the oral pole like its *Podocoryne* orthologue *PcaCnox4* [33], but their *Nematostella* counterpart (*NveAnthox1*) has an aboral expression [34], [37]. Finally, orthologous genes that have similar expression domains are often expressed in different tissues. *PcaCnox1* has an expression in the larva localised at the aboral pole in both the endoderm and the ectoderm [33], but the expression of its orthologue in *Eleutheria EdiCnox5*, although similarly aboral, is restricted to the ectoderm [35].

Thus, current evidence indicates (i) that collinearity of Hox expression is absent in some cnidarian species (e.g. *Clytia hemisphaerica*), implying that a “Hox code” as previously defined for the Bilateria (a positional information along the main body axis specified by a combination of functionally active Hox proteins) is not operating at least in these species, and (ii) that there is no conservation of the expression domains along the oral-aboral axis of orthologous Hox genes among cnidarian species. Cnidarian Hox genes have experienced a wide diversification in their expression sites, with orthologous genes being expressed at different stages during life cycle, in different germ layers, and at different locations, notably with respect to the oral-aboral axis. The evolutionary lability of Hox gene expression sites is further illustrated by the comparison of HOX1 expression at the medusa stage between the two hydrozoan species *Clytia hemisphaerica* and *Podocoryne carnea*: while the *Clytia CheHox1* gene is specically expressed in the sensory cells of the statocysts (this study, figure 2), its orthologue in *Podocoryne PcaCnox1* is expressed in striated muscular cells [24]. Curiously, in cnidarians we are faced with the opposite situation to that observed in bilaterians: while among the later, Hox gene expression along the AP axis is conserved in spite of a tremendous disparity of body plans, their cnidarian orthologues have highly plastic expression territories in animals that share the same basic body plan. Thus, future functional studies in cnidarian models should explore the possibility that, rather than acting in wide range patterning of the body, the cnidarian homologues of the Hox genes might regulate developmental processes at lower (tissue-level and/or cell-level) scales.

An additional conclusion is that Hox genes are inappropriate to decipher body axis homology between bilaterians and cnidarians: for instance they do not tell us which extremity of a cnidarian polyp is homologous to the bilaterian head, if such homology exists. Recent expression studies of cnidarian *Otx* and *Emx*, two transcription factors involved in anterior patterning of the central nervous system in bilaterians, gave similarly unconclusive results with respect to cnidarian/bilaterian “head” homology [54]–[55]. The comparative study of signalling molecules operating in the earliest events of axis specification probably represents a more promising approach to the problem of body axis homology between distantly-related metazoans. Notably, Wnt genes are expressed in staggered domains along the oral-aboral axis in *Nematostella* [56] and in *Clytia* [57], evoking a “Wnt code” [56], [58]–[59]. Based on the position of the Wnt centre, the oral end of cnidarian planulae and polyps seems homologous to the rear end of the Bilateria [60], not to their anterior extremity contrary to earlier claims based on Hox gene expression in the sea anemone [34].

### Origin and early evolution of the ParaHox genes

In the Bilateria, ParaHox genes constitute three groups, GSX (genomic screened homeobox), XLOX (*Xenopus laevis* homeobox 8/insulin promoter factor 1) and CDX (caudal type homeobox), phylogenetically nested within Hox genes. A widely popularised scenario of ParaHox origin postulates that a “ProtoHox” cluster of 2 or 3 genes duplicated into Hox and ParaHox sister-clusters (hypothesis from Brooke [15] also favoured by e.g. [16], [61]–[63]). This scenario was initially proposed based on an unrooted neighbour-joining phylogeny [15] in which GSX, XLOX and CDX sequences appeared as the sister-groups to HOX1/HOX2, HOX3 and HOX9/HOX10 respectively, and on the identification of a ParaHox cluster in the amphioxus genome [15].

Our phylogenetic analysis (figure 1) supports an origin of ParaHox genes by tandem duplications, in agreement with Ryan et al. [37], rather than by duplication of an ancestral “ProtoHox” cluster (figure 4A). In our tree (figure 1), XLOX arises as the sister group of HOX3 as in most previous studies (e.g. [29], [33], [37], [39], [64]–[65]), but GSX and CDX are more closely related to each other than to “anterior” and “posterior” Hox respectively. This topology suggests an independent origin for XLOX and for (GSX+CDX), although we recognise that this scenario is extremely fragile, given the lack of node support, and the notorious instability of GSX position in Hox trees [65]. The important point is that the scenario of a ProtoHox cluster duplication receives no support from phylogenetic analyses. Indeed, only a few previous unrooted neighbour-joining analyses retrieved the (GSX+anterior Hox) and (CDX+posterior Hox) clades [64], [66]–[67], while analyses using an outgroup and/or other reconstruction methods (as in the present study) systematically failed to recover these relations [29], [37], [39], [46], [65], [68]–[70]. In addition, genomic data is not compelling in favour of the cluster duplication scenario, since ParaHox clusters have been identified in only two mammalian species (mouse and human; [67]) in addition to amphioxus, whereas they are absent in other examined deuterostome species [69], [71] and in protostome genomes [72]. The fact that GSX (*NveAnthox2*) and CDX (*NveHD065*) are linked together in the *Nematostella* genome [38] does not represent an argument for an ancient origin of the ParaHox cluster, since it is equally compatible with the scenario favoured by our tree (figure 4A), in which GSX and CDX are sister-genes issued from a duplication independent from the anterior/posterior Hox duplication. Thus, ParaHox clustering might have arisen secondarily in the chordates by intercalation of XLOX between GSX and CDX.

With respect to expression and function, ParaHox genes have been proposed to be implicated in bilaterian antero-posterior patterning of the endoderm in a collinear fashion comparable to Hox genes in the ectoderm [16]. This assumption was based mainly on the spatial and temporal collinearity observed in amphioxus [15] and on the mostly endodermal expression of ParaHox genes observed in amphioxus [15] or in the mouse [73]–[74].

However, the expression of *CheGsx* and *CheCdx* in *Clytia* reported here (figure 3) does not support an ancestral association of ParaHox expression with the endoderm, since both genes are expressed ectodermally, *CheCdx* during development from the blastula stage onwards, and *CheCdx* and *CheGsx* in the medusa tentacle bulbs. In the latter structure, *CheCdx* is probably involved in nematogenesis (the production of the ectodermally located stinging cells), while *CheGsx* expression most likely characterises neuronal precursors.

Our observations in *Clytia* join a wide array of data from other cnidarians and from bilaterians suggesting that ParaHox gene expression is in fact not particularly associated with the endodermal layer. In cnidarians, endodermal GSX expression was only observed in the medusa of *Eleutheria* and in the planula of *Podocoryne* (figure 5; [33], [36]). Neuronal GSX expression, as reported from several hydrozoan and anthozoan species, is clearly more significant. Thus, it has been demonstrated that *Hydra Cnox2* is expressed in bipotent neuronal progenitors giving rise to nematocytes and apical neurons [75], and in anthozoans (*Nematostella* [65] and *Acropora* [32]) *Gsx* expression seems to be restricted to neuronal populations. Since in bilaterians a neural expression of GSX genes is also observed [15], [69]–[70], [76]–[78], GSX is the only Hox-extended gene group showing clear conservation of expression characteristics between cnidarians and bilaterians. Remarkably, GSX is also statistically the best supported group and the only one for which sequence conservation extends outside from the homeodomain [61]. The expression of CDX is similarly not specifically associated with the endoderm. Hence the CDX gene is only expressed in the ectodermal layer in *Clytia* (figure 3) and in *Eleutheria* (figure 5; [35]), whereas it is also exclusively expressed in endoderm in *Nematostella* (figure 5; [37]). Furthermore CDX gene expression is generally extended to the whole posterior end in bilaterians, and not confined to the posterior endoderm (e.g. [15], [70], [79]). Finally, XLOX is the more endoderm-specific ParaHox gene (e.g. [69]–[70], [74]) but this gene has also a neural expression in *Nereis* [80] and *Branchiostoma* [15] and is to date unknown in ecdysozoans and cnidarians.

Finally, collinearity does not seem to be a rule for ParaHox expression. Absence of collinearity is clear for *Clytia CheGsx* and *CheCdx* expression (figure 3), the latter being expressed at both extremities of the planula and the former being undetectable at the same stage. GSX / CDX expression is also clearly not collinear in *Nematostella* (figure 5; [37]). *Eleutheria* is to date the only cnidarian exhibiting collinearity since *EdiCnox4* (CDX) and *EdiCnox2* (GSX) are expressed at opposite poles in the polyp (figure 5; [36]). Among bilaterians, temporal and spatial collinearity was observed in the sea urchin *Strongylocentrotus purpuratus* [69] and the cephalochordate *Branchiostoma floridae* [15] while the polychaete annelid *Nereis virens* displays only spatial collinearity [80] and ParaHox genes are expressed in a non-collinear manner in the urochordate *Ciona intestinalis* [71] and in the polychaete annelid *Capitella* sp. I [70]. Altogether, these data seem to exclude an ancient role for ParaHox genes in patterning the endoderm along the main body axis.

### Conclusions

Our analyses of the Hox-extended family sequences and expression patterns in the hydrozoan *Clytia hemisphaerica* confronted with available data from other cnidarian species and from the Bilateria led us to reassess the early evolution of Hox and Hox-related genes family. Hox / ParaHox gene implication in axial patterning does not appear as a conserved feature among cnidarians and the Hox code seems more likely to be an innovation of the Bilateria (in agreement with Kamm et al. [35]). Hox/ParaHox paralogous groups underwent diverging histories among cnidarian lineages, both in terms of gene duplications and losses, and in terms of gene expression, probably reflecting diversification of functions.

Even if not particularly associated with axial patterning, transcription factors of the Hox-extended family probably played important roles in the evolution and diversification of the body plan during cnidarian evolution, through extensive gene co-option. Notably, they were probably involved in shaping the medusa (clearly a modified body plan derived from within the cnidarians), as suggested by HOX1 gene expression in mechanosensory cells of the statocysts (in *Clytia*, figure 2P) or in striated muscular cells (in *Podocoryne* [24]), and by the restricted expression of Clytia *CheMox* in particular areas of the medusa gastro-vascular system. Future progress in our understanding of the significance of Hox/ParaHox family genes for cnidarian and eumetazoan body plan evolution will require data from understudied cnidarian classes (Scyphozoa, Cubozoa, Staurozoa), as well as more experimental work, including gene surexpression / inactivation studies and the characterisation of target genes, in order to determine the exact roles of these transcription factors in cnidarian development and morphogenesis.

## Materials and Methods

### Animals

Colonies of *Clytia hemisphaerica* were cultured in the laboratory and polyps, medusa, eggs, embryos and larva were obtained as previously described [81].

### *Clytia* cDNA library and ESTs

Hox-related sequences were retrieved from a collection of 80.000 EST (Expressed Sequenced Tag) sequences generated from a mixed-stage normalised cDNA library as previously described [81]. EST sequencing was performed at the Genoscope (Evry, France).

### Hox-related sequence identification

A systematic search for sequences of the Antp super-class was performed on the *Clytia* ESTs. The identification was based on sequence similarity in the Antp-homeodomain as revealed by BLAST searching (tBLASTn with a 1e^−7^ expected value threshold) with representatives from all known Antp sub-classes (Hox-extended, BarH, Dlx, Emx, Hlx, NK, Tlx) from *Nematostella* [41] and *Drosophila*.

### Phylogenetic analysis

Homeodomain sequences from *Clytia* were aligned with sequences from a wide range of cnidarian and bilaterian homeodomains obtained by BLAST search in the GenBank and CnidBase databases. In the present study, the adopted strategy was to maximise taxonomic sampling among the cnidarians in order to have a representative view of their diversity and to allow discussions about Hox/ParaHox evolution within cnidarian lineages. Thus a matrix was built with 117 cnidarian and 94 bilaterian Antp homeodomain sequences and was completed by 5 sequences from the placozoan *Trichoplax adherens* [46], the 8 available sequences from the demosponge *Amphimedon queenslandica* [47] and the 4 available sequences from the ctenophore *Mnemiopsis leidyi* [48].

The cnidarian dataset included the full Antp complements from *Nematostella vectensis* [41]. The matrix was completed with published Hox and Hox-related complete or near complete homeodomain sequences from three main cnidarian groups: the anthozoans *Acropora millepora* [32], [82] and *Metridium senile* [26], the scyphozoan *Cassiopeia xamachana* [28] and the hydrozoans *Eleutheria dichotoma* (Capitata, [25]), *Podocoryne carnea* (Filifera, [31], [33]), *Hydractinia symbiolongicarpus* (Filifera, [27], [83]) and several Hydra species (*Hydra magipapillata*, *Hydra vulgaris*, *Hydra viridis* formerly *Chlorohydra viridissima* (Aplanulata), [22], [29], [38]). These sequences were named after their published names and for *Nematostella* Hox sequences, for which several names have been proposed, after the nomenclature used by Ryan et al. [37]. The bilaterian data set comprised sequences from representative species of Deuterostomia (the Antp complement of *Branchiostoma floridae*, and one Not sequence from *Gallus gallus*), Ecdysozoa (the Antp complement from *Drosophila melanogaster* and one Hox3 sequence from *Cupiennius salei*) and Lophotrochozoa (*Nereis virens* Antp sequences, one Xlox sequence from *Capitella sp.*, one Xlox sequence from *Euprymna scolopes* and one Mox sequence from *Haliotis rufescens*). Accession numbers are available in figure S3.

In this study, we have chosen an outgroup including all Antp non-Hox/ParaHox sequences known from the included species. Indeed, our preliminary phylogenetic analyses have shown that the internal topology is very sensitive to rooting sequence selection and the only way to avoid rooting bias was to perform a global phylogeny of the Antp homeodomains (see full tree with non-compressed outgroup in Additional file 1).

ML analysis was performed using PhyML [84] with the JTT amino acid substitution model, 8 categories of substitution rates with an estimated Gamma distribution parameter and an estimated proportion of invariable sites. Statistical support was evaluated by 100 replicates of bootstrap. NJ analysis was performed using PAUP4.0b10 [85] uncorrected distance. Statistical support for the NJ topology was assessed by 1000 bootstrap replicates.

### *In Situ* Hybridisation

DIG-labelled antisense RNA probes synthesis, samples fixation and *in situ* hybridisation were carried out as previously described [81], except for colour development which was performed using BM purple reagent (Roche). After postfixation 30 min. in 4% paraformaldehyde/PBStween, the nuclei were stained with Dapi (1 µg/ml) during 15–30 min followed by several washes in PBStween. Samples were then mounted in 60% glycerol/PBS. Double *in situ* hybridisation were performed as previously described [44].

### Immunostaining

Animals were fixed in 4% paraformaldehyde in PBS (10 mM Na2HPO4, 150 mMNaCl, pH 7.5) for 15 min, at room temperature, then samples were washed several times in PBS, dehydrated through a graded series of ethanol and stored in methanol at −20°C. After stepwise re-hydration to PBS, samples were permeabilised with Triton-X100 (0.2% in PBS, then 0.01% in PBS, 10 min at room temperature). After blocking with 1% bovine serum albumin, samples were incubated with the following primary antibodies for 2 hours at room temperature: rabbit polyclonal anti-FMRFamide (ABcam, 1/1000) and mouse monoclonal anti-acetylated α-tubulin (6-11-B1, Sigma, 1/1000). After washing in PBS triton-X100 0.01% solution (PBST), samples were incubated overnight at 4°C with the following secondary antibodies (1/1500): Alexa Fluor ® 568 goat anti-rabbit IgG or Alexa Fluor ® 488 goat anti-mouse IgG (Molecular probes). Primary and secondary antibodies were diluted in 1× PBS containing 0.01% Triton-X100. Samples were stained finally with DAPI (1 µg/ml) for 15 mn, in PBST and mounted in Vectashield ® solution.

### Imaging

Fluorescence and most DIC images were acquired with an Olympus BX61 microscope using a Q-imaging Camera with Image Pro plus software ® (Mediacybernetics).

## Supporting Information

Figure S1 Phylogenetic relationships between cnidarian, placozoan and bilaterian Hox/ParaHox related homeodomains inferred by ML analysis. Same tree as figure 1 but with a non-compressed outgroup. Numbers above branch indicate percentages of 100 bootstrap replicates in the ML analysis. Abbreviations: Afo, Acropora formosa; Ami, Acropora millepora; Aqu, Amphimedon queenslandica; Bfl, Branchiostoma floridae; Che, Clytia hemisphaerica; Csa, Cupiennus salei; Csp, Capitella sp.; Cvi, Chlorohydra viridissima; Cxa, Cassiopeia xamachana; Dme, Drosophila melanogaster; Edi, Eleutheria dichotoma; Esc, Euprymna scolopes; Gga, Gallus gallus; Hma, Hydra magnipapillata; Hru, Haliotis rufescens; Hsy, Hydractinia symbiolongicarpus; Hvu, Hydra vulgaris ; Mle, Mnemiopsis leidyi; Mse, Metridium senile; Mus, Mus musculus; Ner, Nereis virens; Nve, Nematostella vectensis; Pca, Podocoryne carnea; Tad, Trichoplax adhaerens.
(2.12 MB JPG)

Figure S2 Phylogenetic relationships between cnidarian, placozoan and bilaterian Hox/ParaHox related homeodomains inferred by NJ analysis. The analysis was performed on the same alignment as for the ML analysis. Numbers above branch indicate percentages of 1000 bootstrap replicates in the NJ analysis. Abbreviations: Afo, Acropora formosa; Ami, Acropora millepora; Aqu, Amphimedon queenslandica; Bfl, Branchiostoma floridae; Che, Clytia hemisphaerica; Csa, Cupiennus salei; Csp, Capitella sp.; Cvi, Chlorohydra viridissima; Cxa, Cassiopeia xamachana; Dme, Drosophila melanogaster; Edi, Eleutheria dichotoma; Esc, Euprymna scolopes; Gga, Gallus gallus; Hma, Hydra magnipapillata; Hru, Haliotis rufescens; Hsy, Hydractinia symbiolongicarpus; Hvu, Hydra vulgaris; Mle, Mnemiopsis leidyi; Mse, Metridium senile; Mus, Mus musculus; Ner, Nereis virens; Nve, Nematostella vectensis; Pca, Podocoryne carnea; Tad, Trichoplax adhaerens.
(2.36 MB JPG)

Figure S3 Accession numbers of sequences used for phylogenetic analyses
(0.07 MB PDF)
