# Supplementary figures and images for: Are Hox Genes Ancestrally Involved in Axial Patterning? Evidence from the Hydrozoan Clytia hemisphaerica (Cnidaria)
Source: PLoS One. 2009 Jan 21;4(1):e4231. doi: 10.1371/journal.pone.0004231 (PMC2626245; doi:10.1371/journal.pone.0004231)

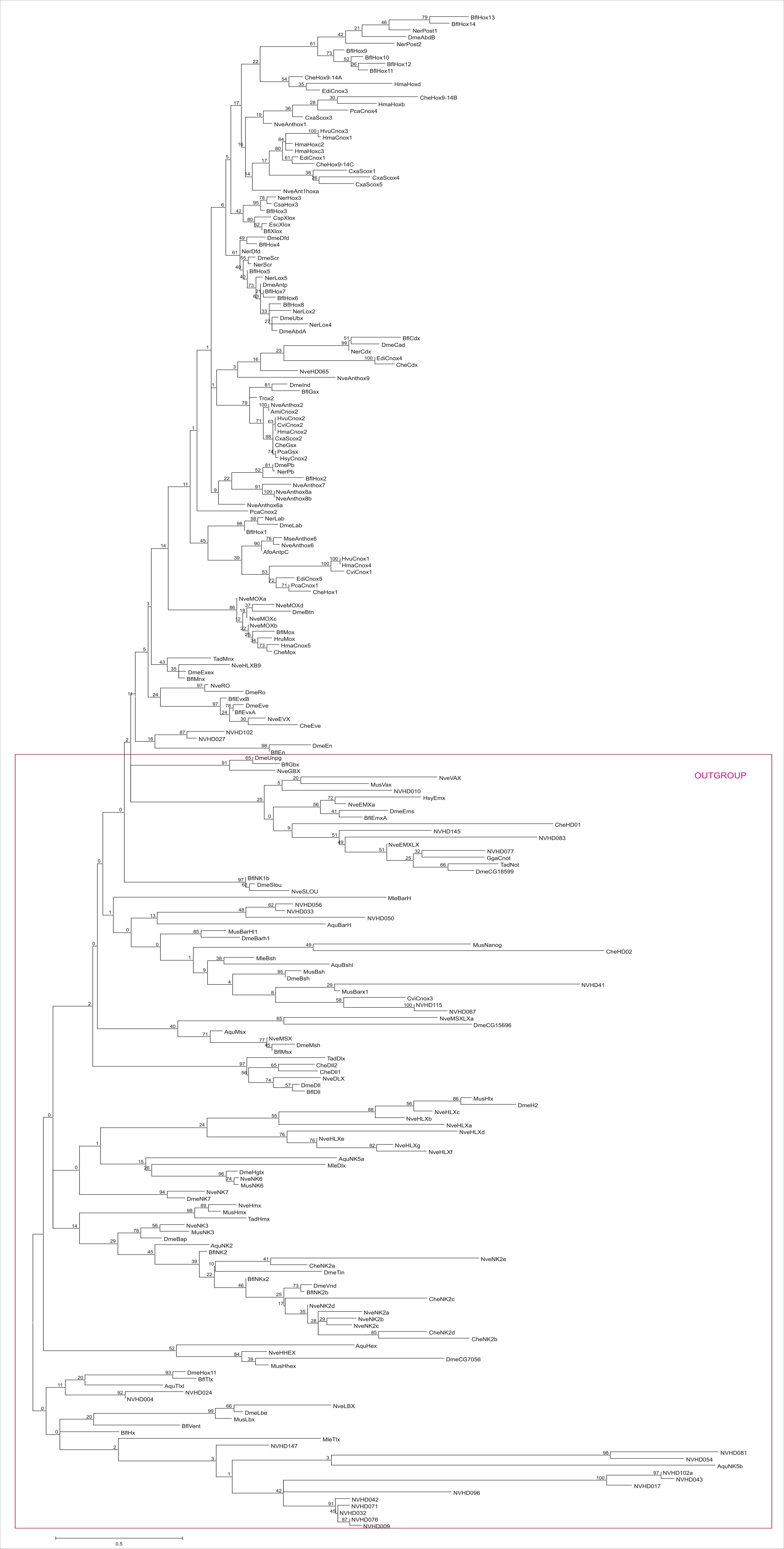

Supplement: Figure S1 — Phylogenetic relationships between cnidarian, placozoan and bilaterian Hox/ParaHox related homeodomains inferred by ML analysis. Same tree as figure 1 but with a non-compressed outgroup. Numbers above branch indicate percentages of 100 bootstrap replicates in the ML analysis. Abbreviations: Afo, Acropora formosa; Ami, Acropora millepora; Aqu, Amphimedon queenslandica; Bfl, Branchiostoma floridae; Che, Clytia hemisphaerica; Csa, Cupiennus salei; Csp, Capitella sp.; Cvi, Chlorohydra viridissima; Cxa, Cassiopeia xamachana; Dme, Drosophila melanogaster; Edi, Eleutheria dichotoma; Esc, Euprymna scolopes; Gga, Gallus gallus; Hma, Hydra magnipapillata; Hru, Haliotis rufescens; Hsy, Hydractinia symbiolongicarpus; Hvu, Hydra vulgaris ; Mle, Mnemiopsis leidyi; Mse, Metridium senile; Mus, Mus musculus; Ner, Nereis virens; Nve, Nematostella vectensis; Pca, Podocoryne carnea; Tad, Trichoplax adhaerens. (2.12 MB JPG) [file pone.0004231.s001.jpg]

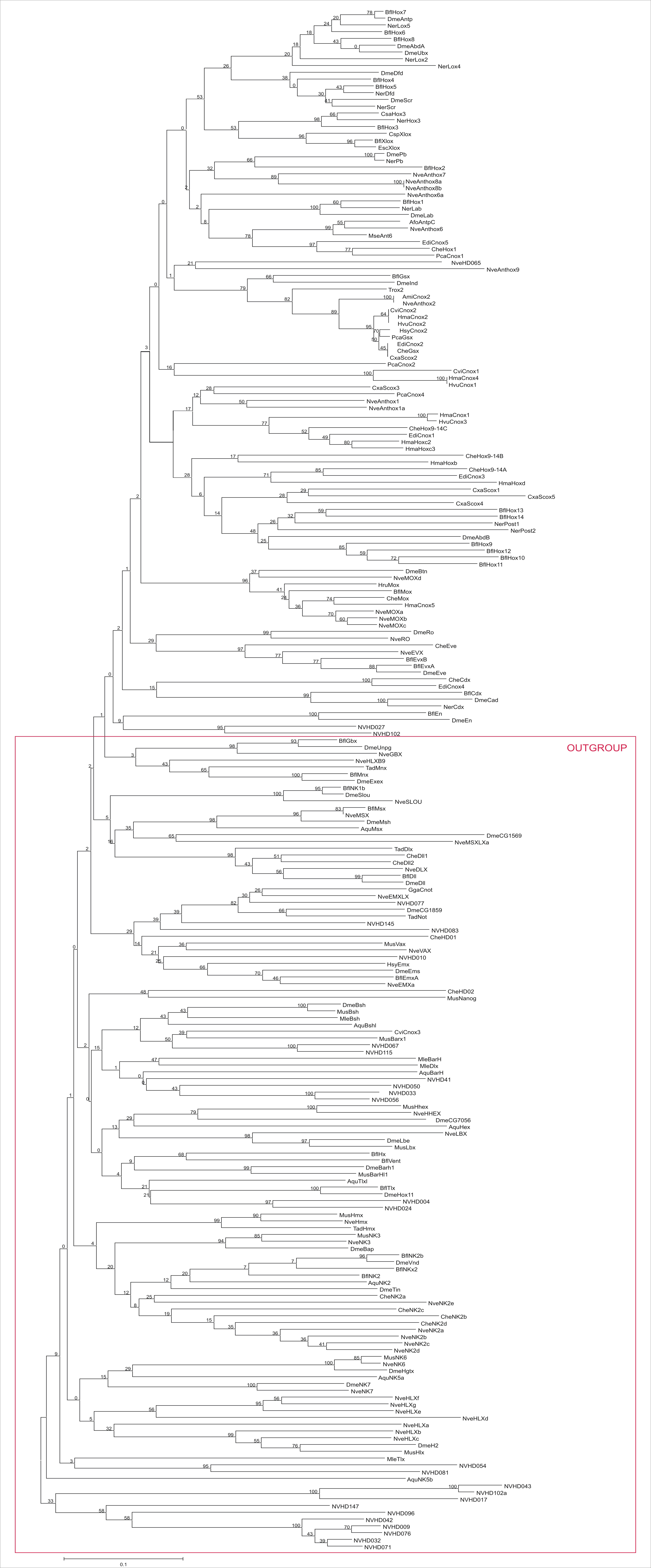

Supplement: Figure S2 — Phylogenetic relationships between cnidarian, placozoan and bilaterian Hox/ParaHox related homeodomains inferred by NJ analysis. The analysis was performed on the same alignment as for the ML analysis. Numbers above branch indicate percentages of 1000 bootstrap replicates in the NJ analysis. Abbreviations: Afo, Acropora formosa; Ami, Acropora millepora; Aqu, Amphimedon queenslandica; Bfl, Branchiostoma floridae; Che, Clytia hemisphaerica; Csa, Cupiennus salei; Csp, Capitella sp.; Cvi, Chlorohydra viridissima; Cxa, Cassiopeia xamachana; Dme, Drosophila melanogaster; Edi, Eleutheria dichotoma; Esc, Euprymna scolopes; Gga, Gallus gallus; Hma, Hydra magnipapillata; Hru, Haliotis rufescens; Hsy, Hydractinia symbiolongicarpus; Hvu, Hydra vulgaris; Mle, Mnemiopsis leidyi; Mse, Metridium senile; Mus, Mus musculus; Ner, Nereis virens; Nve, Nematostella vectensis; Pca, Podocoryne carnea; Tad, Trichoplax adhaerens. (2.36 MB JPG) [file pone.0004231.s002.jpg]
