# Supplementary material for: Are Hox Genes Ancestrally Involved in Axial Patterning? Evidence from the Hydrozoan Clytia hemisphaerica (Cnidaria)
Source: PLoS One. 2009 Jan 21;4(1):e4231. doi: 10.1371/journal.pone.0004231 (PMC2626245; doi:10.1371/journal.pone.0004231)
Supplement: Figure S3 — Accession numbers of sequences used for phylogenetic analyses (0.07 MB PDF) [file pone.0004231.s003.pdf]

| Species | Gene | Accession |
|---|---|---|
| *Acropora formosa* | AfoAntpC | S36771 |
| *Acropora millepora* | AmiCnox2 | AAK28380.1 |
| *Amphimedon queenslandica* | AquBarH | ACC76765.1 |
| | AquBshl | ACA04743.1 |
| | AquHex | ACC76761.1 |
| | AquMsx | ACC76762.1 |
| | AquNK2 | ACA04745.1 |
| | AquNK5a | ACC76764.1 |
| | AquNK5b | ACC76763.1 |
| | AquTlxl | ACA04744.1 |
| *Branchiostoma floridae* | BflCdx | AAC39017.1 |
| | BflDll | P53772.1 |
| | BflEmxA | AAK93792.1 |
| | BflEn | AAB40144.1 |
| | BflEvxA | AF374191_1 |
| | BflEvxB | AF374192_1 |
| | BflGbx | ABD85192.1 |
| | BflGsx | AAC39015.1 |
| | BflHox1 | CAA84514.1 |
| | BflHox10 | CAA84522.1 |
| | BflHox11 | AAF81909.1 |
| | BflHox12 | AAF81903.1 |
| | BflHox13 | AF276815_1 |
| | BflHox14 | AAF81905.1 |
| | BflHox2 | BAA78621.1 |
| | BflHox3 | CAA48180.1 |
| | BflHox4 | BAA78622.1 |
| | BflHox5 | CAA84517.1 |
| | BflHox6 | ABX39490.1 |
| | BflHox7 | ABX39491.1 |
| | BflHox8 | ABX39492.1 |
| | BflHox9 | ABX39493.1 |
| | BflHx | AF303216_1 |
| | BflMnx | AF308821_1 |
| | BflMox | AF490355_1 |
| | BflMsx | CAA10201.1 |
| | BflNK1b | CAD83854.1 |
| | BflNK2 | AF482469_1 |
| | BflNK2b | AAD01958.1 |
| | BflNKx2 | AAC35350.1 |
| | BflTlx | CAD83853.1 |
| | BflVent | AF303217_1 |
| | BflXlox | AAC39016.1 |
| *Clytia hemisphaerica* | CheCdx | FJ392842 |
| | CheDll1 | FJ392843 |
| | CheDll2 | FJ392844 |
| | CheEve | FJ392845 |
| | CheGsx | FJ392846 |
| | CheHD01 | FJ392847 |
| | CheHD02 | FJ392848 |
| | CheHox1 | FJ392849 |
| | CheHox9-14A | FJ392850 |
| | CheHox9-14B | FJ392851 |

| | | |
|---|---|---|
| | CheHox9-14C | FJ392852 |
| | CheMox | FJ392853 |
| | CheNK2a | FJ392854 |
| | CheNK2b | FJ392855 |
| | CheNK2c | FJ392856 |
| | CheNK2d | FJ392857 |
| *Cupiennus salei* | CsaHox3 | AJ005643.1 |
| *Capitella sp.* | CspXlox | AAZ95509.1 |
| *Hydra viridis* | CviCnox1 | CAA45908.1 |
| | CviCnox2 | CAI38829.1 |
| | CviCnox3 | CAA45910.1 |
| *Cassiopea xamachana* | CxaScox1 | AF124591_1 |
| | CxaScox2 | AF124592_1 |
| | CxaScox3 | AF124593_1 |
| | CxaScox4 | AF124594_1 |
| | CxaScox5 | AF124595_1 |
| *Drosophila melanogaster* | DmeAbdA | P29555 |
| | DmeAbdB | P09087 |
| | DmeAntp | P02833 |
| | DmeBap | P22809 |
| | DmeBarh1 | Q24255 |
| | DmeBsh | Q04787 |
| | DmeBtn | AAF56025.1 |
| | DmeHox11 | XP_001993871.1 |
| | DmeCad | P09085 |
| | DmeCG15696 | DQ138716.1 |
| | DmeCG18599 | NM_142444.1 |
| | DmeCG7056 | NM_142681.1 |
| | DmeDfd | P07548 |
| | DmeDll | P20009 |
| | DmeEms | P18488 |
| | DmeEn | P02836 |
| | DmeEve | P06602 |
| | DmeExex | NM_139907.2 |
| | DmeH2 | P10035 |
| | DmeHgtx | NM_144357.2 |
| | DmeInd | NM_206365.1 |
| | DmeLab | P10105 |
| | DmeLbe | NM_079711.2 |
| | DmeMsh | Q03372 |
| | DmeVnd | P22808 |
| | DmeNK7 | NM_169580.1 |
| | DmePb | P31264 |
| | DmeRo | P10181 |
| | DmeScr | P09077 |
| | DmeSlou | P22807 |
| | DmeTin | P22711 |
| | DmeUbx | P83949 |
| | DmeUnpg | NM_057798.2\| |
| *Eleutheria dichotoma* | EdiCnox1 | ABE68629.1 |
| | EdiCnox2 | AAB20573 |
| | EdiCnox3 | AAB48008.1 |
| | EdiCnox4 | ABE68631.1 |
| | EdiCnox5 | AAB48010.1 |

| | | |
|---|---|---|
| *Euprymna scolopes* | EscXlox | ABD16192.1 |
| *Gallus gallus* | GgaCnot | AAB00687.1 |
| *Hydra magnipapillata* | HmaCnox1 | CAB88385.1 |
| | HmaCnox2 | AJ277388.1 |
| | HmaCnox4 | pirS39067 |
| | HmaCnox5 | Z22640 |
| | HmaHoxb | Chourrout et al. 2006 |
| | HmaHoxc2 | Chourrout et al. 2006 |
| | HmaHoxc3 | Chourrout et al. 2006 |
| | HmaHoxd | Chourrout et al. 2006 |
| *Haliotis rufescens* | HruMox | CAA53027.1 |
| *Hydractinia symbioloncarpus* | HsyCnox2 | ABC00746.1 |
| | HsyEmx | CAA72534.1 |
| *Hydra vulgaris* | HvuCnox1 | CAB88384.1 |
| | HvuCnox2 | CAB87555.1 |
| | HvuCnox3 | CAB88385.1 |
| *Mnemiopsis leidyi* | MleBarH | ACD85818.1 |
| | MleBsh | ACD85817.1 |
| | MleDlx | ACD85819.1 |
| | MleTlx | ACD85820.1 |
| *Metridium senile* | MseAnthox6 | AY096246.1 |
| *Mus musculus* | MusBarHl1 | P63157 |
| | MusBarx1 | Q9ER42 |
| | MusBsh | NM_178245.3 |
| | MusHhex | P43120 |
| | MusHlx | Q61670 |
| | MusHmx | P43687 |
| | MusLbx | P52955 |
| | MusNanog | XM_001471588.1 |
| | MusNK3 | NM_010921.3 |
| | MusNK6 | XM_001474383.1 |
| | MusVax | Q9WTP9 |
| *Nereis virens* | NerCdx | AAN11403.2 |
| | NerDfd | AAD46169.2 |
| | NerHox3 | AAD46168.1 |
| | NerLab | AAD46166.2 |
| | NerLox2 | AAD46171.2 |
| | NerLox4 | AF151669 |
| | NerLox5 | AAD46174.2 |
| | NerPb | AAD46167.2 |
| | NerPost1 | AAD46175.1 |
| | NerPost2 | AAD46176.2 |
| | NerScr | AF151667 |
| *Nematostella vectensis* | NveAnthox1 | DQ206287.1 |
| | NveAnthox1a | DQ206337.1 |
| | NveAnthox6 | DQ206301.1 |
| | NveAnthox6a | DQ206321.1 |
| | NveAnthox7 | DQ206311.1 |
| | NveAnthox8a | DQ206299.1 |
| | NveAnthox8b | DQ315389.1 |
| | NveAnthox9 | DQ206290.1 |
| | NveDLX | DQ206283.1 |
| | NveEMXa | DQ206302.1 |
| | NveEMXLX | DQ206278.1 |
| | NveEvx | DQ206338.1 |

| | |
|---|---|
| NveGBX | DQ206305.1 |
| NveGSX | DQ206295.1 |
| NveHHEX | DQ206317.1 |
| NveHLXa | DQ206331.1 |
| NveHLXb | DQ206303.1 |
| NveHLXB9 | DQ206324.1 |
| NveHLXc | DQ206333.1 |
| NveHLXd | DQ206343.1 |
| NveHLXe | DQ206282.1 |
| NveHLXf | DQ206318.1 |
| NveHLXg | DQ206314.1 |
| NveHmx | DQ206341.1 |
| NveLBX | DQ206291.1 |
| NveMOXa | DQ206313.1 |
| NveMOXb | DQ206339.1 |
| NveMOXc | DQ206306.1 |
| NveMOXd | DQ206342.1 |
| NveMSX | DQ206296.1 |
| NveMSXLXa | DQ206269.1 |
| NveNK2a | DQ206320.1 |
| NveNK2b | DQ206262.1 |
| NveNK2c | DQ206270.1 |
| NveNK2d | DQ206285.1 |
| NveNK2e | DQ206310.1 |
| NveNK3 | DQ206336.1 |
| NveNK6 | DQ206340.1 |
| NveNK7 | DQ206276.1 |
| NveRO | DQ206293.1 |
| NveSLOU | DQ206255.1 |
| NveVAX | DQ206277.1 |
| NVHD004 | DQ206223.1 |
| NVHD009 | DQ206261.1 |
| NVHD010 | DQ206300.1 |
| NVHD017 | DQ206304.1 |
| NVHD024 | DQ206224.1 |
| NVHD027 | DQ206263.1 |
| NVHD032 | DQ206266.1 |
| NVHD033 | DQ206312.1 |
| NVHD042 | DQ206315.1 |
| NVHD043 | DQ206268.1 |
| NVHD050 | DQ206319.1 |
| NVHD054 | DQ206272.1 |
| NVHD056 | DQ206274.1 |
| NVHD065 | DQ301955.1 |
| NVHD067 | DQ206323.1 |
| NVHD071 | DQ206325.1 |
| NVHD076 | DQ206328.1 |
| NVHD077 | DQ206329.1 |
| NVHD081 | DQ206330.1 |
| NVHD083 | DQ206279.1 |
| NVHD096 | DQ206334.1 |
| NVHD102 | DQ206284.1 |
| NVHD115 | DQ206289.1 |
| NVHD145 | DQ206346.1 |
| NVHD147 | DQ206297.1 |

| | | |
|---|---|---|
| | NVHD41 | DQ206267.1 |
| *Podocoryne carnea* | PcaCnox1 | CAA57211.1 |
| | PcaCnox2 | BAA94091.1 |
| | PcaCnox4 | AAK63185.1 |
| | PcaGsx | AAG09805.1 |
| *Trichoplax adhaerens* | TadDlx | ACH57166.1 |
| | TadHmx | ACH57163.1 |
| | TadMnx | ACH57159.1 |
| | TadNot | ACH57165.1 |
| | Trox2 | XP_002118201.1 |
